# Regional Injury Classification and Treatment of Open Pelvic Fractures

**DOI:** 10.1111/os.12554

**Published:** 2019-11-15

**Authors:** Zheng‐hao Wang, Kai‐nan Li

**Affiliations:** ^1^ Department of Orthopaedic Surgery Affiliated Hospital of Chengdu University Chengdu Sichuan China

**Keywords:** Classification, Combined injury, Fracture, Openness, Pelvis, Mortality

## Abstract

**Objectives:**

To propose the regional injury classification of open pelvic fracture and summarize the characteristics of its treatment.

**Methods:**

Clinical data for 67 open pelvic fractures treated from January 2001 to December 2017 were retrospectively analyzed. There were 48 male and 19 female patients. The patients were aged from 12 to 68 years old, and their pelvic fractures were categorized according to Tile classification (type A: 23 cases; type B: 19 cases; type C: 25 cases). Main injury distribution: pubic perineum, 29 cases; ilioinguinal, 20 cases; and sacroiliac, 7 cases. There were 5 cases of ilioinguinal‐perineum and 6 cases of sacroiliac‐perineum injury. Based on the region of the injury, the mortality and combined injury of each group were observed. The relationship between regional injury groups and death was examined.

**Results:**

Following active treatment, 28 patients died. The mortality rate was 41.8% (28/67), with 39 patients surviving. The average follow‐up time was 6 months (3 months to 1 year after discharge). Majeed pelvic fracture score: the score was excellent in 12 cases, good in 14 cases, fair in 9 cases, and poor in 4 cases; there was an excellent and good rate of 66.7%. Open pelvic fracture regional injury classification includes: the perineal type (type I); the ilioinguinal type (type II); the sacroiliac type (type III); and the composite type (type IV). All types were independent of each other, and the mortality of open pelvic fractures was positively correlated with this classification, with a correlation coefficient of 0.620 (*P* = 0.001 < 0.05; the difference was statistically significant). In this study, cystourethral injury, anorectal injury, and infection were the main combined injuries of type I. The type II and III injuries were mainly iliac vascular injuries. The main combined injuries of type IV were infection, injury of ilium vessels and its branches. There was a statistical difference among the combined injuries of each subtype (*P* = 0.001 < 0.05).

**Conclusions:**

The criteria for regional classification were clear, the mortality of the four subtypes increased gradually, and the incidence of combined injury of each subtype varied. Each subtype had different therapeutic characteristics.

## Introduction

Open pelvic fractures are severe and fatal traumas, with a lower incidence of approximately 2%–4% of pelvic fractures (4%–5% of total fractures), but the mortality rate is as high as 30%–50%, with serious complications, often associated with multiple injuries^1^, ^2^. At present, there is no expert consensus on the classification of open pelvic fractures. However, there is a great need for a scientific and effective classification method, to make timely assessment of the severity of open pelvic fractures and to guide the correct formulation of treatment plans^3^. At present, there are three kinds of classification methods reported in local and foreign literature. The first type, Hanson classification, combines the wound classification of open fractures with the classification method of pelvic fractures, taking into account soft tissue and pelvic organ injuries^4^. This classification only takes into account the type of fracture, age, the injury mechanism, and the soft tissue injury. However, shock, iliac vascular injury, bladder and urethral injury, anus and intestinal injury, and other serious combined injuries were not considered^5^, ^6^. This classification does not guide clinical treatment. For the second type, Jones–Powell typing on the basis of Hanson typing, the pelvic stability was studied, but there was a risk of oversimplification, especially when the injury of peripelvic soft tissue and its viscera was simply classified as a rectal and perineal injury^7^. The third type is the Bircher classification, which takes into account the Tiles pelvic fracture classification, pelvic organ, including vaginal, rectal and urinary system, injuries, and associated soft tissue injuries, but fails to include the subdivision of soft tissue injuries^8^. The classification standard of each subtype is unclear, especially when the difference between the three groups is small. The Bircher classification is relatively scientific, but the classification system is too complicated and difficult to remember, and it is inconvenient to apply in practice. The fourth type, the Faringer classification, is based on the injury region, with the injury area used as a classification criterion for the first time, but the relationship between classification and mortality has not been clearly discussed, and there is still a lack of evaluation of prognosis^9^. At the same time, in clinical practice, we found that there are cases with injuries in two or more regions at the same time, so it is necessary to divide and evaluate the injuries separately. In reviewing the above four classification methods, the authors found that none of the above types clearly identified the key causes of death and significant combined injuries of open pelvic fractures, and could not timely and accurately guide the emergency treatment of clinicians. The above classification has the potential to result in misdiagnosis and missed treatment. There are many factors affecting the mortality of open pelvic fractures, and it is of great importance to investigate the combined injuries related to patient deaths^10^. The clinical data for 67 cases of open pelvic fractures admitted and treated in our hospital from January 2001 to December 2017 were reviewed and summarized. A new type of open pelvic fracture classification, regional injury classification, was proposed to achieve the following purposes: (i) to identify the differences of regional injury types of open pelvic fractures, and to explore the correlation between the causes of death and combined injuries in each type; (ii) to explore the treatment characteristics of each type, and to formulate the flowchart of treatment for open pelvic fractures; and (iii) to analyze and summarize the advantages and disadvantages of this regional injury classification.

## Materials and Methods

### *Inclusion and Exclusion Criteria*

Inclusion criteria: (i) cases of initial open pelvic fracture; (ii) patients admitted to hospital or transferred within 24 h from the scene of the injury; and (iii) complete case data and follow‐up materials. Exclusion criteria: (i) perforating injuries such as gunshot or knife stab wounds; (ii) closed pelvic fracture; (iii) patients with incomplete data as a result of transfer, automatic departure, and loss of follow up; and (iv) history of pelvic fracture surgery.

### *Materials*

A total of 67 patients were included in this study, 48 males and 19 females, aged from 12 to 68 years, with an average of 34 years. Cause of injury: 33 traffic injuries, 19 mechanical injuries, and 15 falling injuries. Soft tissue injuries classified by Gustilo–Anderson^11^: type III, 67 cases. Fracture Tile type^12^: type A, 23 cases (A2: 20 cases), accounting for 37.5% (21/56); type B, 16 cases, accounting for 28.3% (19/67); and type C, 25 cases, accounting for 37.3% (25/67). Main wound distribution: pubic perineum, 29 cases; ilioinguinal, 20 cases; sacroiliac, 7 cases. There were 6 cases of ilioinguinal‐perineum and 5 cases of sacroiliac‐perineum injury. The injury severity score (ISS) ranged from 25 to 48, with an average of 34.6 points. All 67 cases were complicated with shock; 40 cases (59.7% or 40/67) were combined with iliac vascular injury, 42 cases (62.7% or 42/67) with bladder urethral injury (17 cases with simple bladder rupture), 29 cases (43.3% or 29/67) with anal and intestinal injury, 21 cases (31.3% or 21/67) with visceral injury of the liver, spleen and kidney, and 26 cases (38.8% or 26/67) with limb fracture.

### *Treatment Method*

The 67 patients in this study underwent multiple surgical treatment, with an average of five operations per patient. Hemostasis of massive hemorrhage was performed in 17 cases with ligation or embolization of the internal iliac artery, and in 20 cases with wound channel and retroperitoneal packing for hemostasis. For pelvic fractures, only external fixation was performed. External colostomy and cystostomy were performed for 38 cases of pelvic organ (colorectal and cystourethral) injuries, and ligation of the distal and proximal ruptured intestinal canal was performed for emergency cases, and resuscitation was performed in ICU after surgery. Pelvic fixation, urethral reconstruction, and colon retraction await after stable condition anaphase treatment. Internal fixation was performed in 20 cases of limb closed fracture injuries, and simple external fixation was performed in 6 cases of limb open fracture injuries. For the primary operation, iliac artery ligation was carried out in 15 cases, with 2 cases of selective internal iliac arteriography embolization after surgery; there were hemostasis through wound tract and retroperitoneal tamponade in 20 patients, and 12 cases of postoperative additional wound filling. Bladder fistula occurred in 16 cases and bladder repair plus urethral reconstruction in 6 cases. A total of 5 cases of intra‐peritoneal colorectal injuries were repaired in a one‐stage operation, and 2 cases were treated with ostomy. There were 15 cases of epiperitoneal rectal injuries treated with colostomy. In 3 patients with rectal injury, the distal and proximal intestines were clamped by emergency forceps. After the condition was stable, the intestines were anastomosed surgically. Among the concomitant injuries, there were 21 cases of other abdominal organ injuries: 10 cases of liver rupture, 7 cases of spleen rupture, and 4 cases of kidney rupture. Nineteen cases of ruptured jejunum, ileum, and mesenteric vessels were treated by laparotomy. Among these cases, there were 5 cases of liver tamponade, 7 cases of splenectomy, 3 cases of renal resection, and 1 case of postoperative selective renal angiography embolization. There was no special treatment for 3 cases of craniocerebral injury and 7 cases of spinal fracture with paraplegia in emergency departments. Thoracic duct placement was observed in 3 of 5 cases with chest injury; 17 cases of skin avulsion injury with defects were treated with perforation and relaxation suture. Emergency treatment to restore the stability of the pelvic ring. Pelvic fixation was performed within 7 to 10 days after injury, and external fixation was continued when the infection was severe. After the wound was closed and stable and the infection was controlled, the patients with obvious fracture displacement and an unstable pelvic ring were treated with internal fixation. After cystectomy and sigmoidostomy, the patients underwent bladder repair, urethra reconstruction, and colonization reconstruction.

The patients underwent emergency external fixation after their injury. Under general anesthesia, the patients lay on their backs and underwent continuous supracondylar traction of the femoral bone, and the sacroiliac joint was well reduced by fluoroscopy. The insertion point of the Schanz needle was marked with a marker, and 2‐7 cm or two adjacent points above the anterior inferior ilium spine and the bilateral anterior superior ilium spine were taken as entry points, and bilateral femoral arteries were marked. An approximate 1‐cm incision was made according to the marking point, and the soft tissue was passively separated vertically to the bone cortex to avoid injury or traction of peripheral nerves and blood vessels. The flat head soft tissue protection sleeve was placed on the bone surface of the incision entry point; the bone cortex was drilled, the hole was not expanded, and the Schanz needle, with a diameter of 4.0 or 5.0 mm, was directly inserted in the direction of the nail. After nail placement, the screw placement was monitored by modified bilateral pelvic outlet position and bilateral ilium oblique fluoroscopy. The connecting rod and clamp were installed on the same side as the external fixing frame and locked in the shape of "\ /"; the far end was connected by a single transverse connecting rod, and the proximal end was connected by a single transverse bar or two connecting rods approximately 140° to the front of the abdomen. The fixation clip was locked after the reduction of the pelvis. The reduction effect was determined by fluoroscopy, the incision beside the Schanz needle was sutured in turn, and the aseptic excipients were bandaged under pressure.

### *Research Methods*

The 67 patients with open pelvic fractures were divided into four groups according to the different distribution of injuries. The mortality and combined injuries of each group were assessed, and clear associations identified between grouping and mortality. The combined injuries in the four groups included shock, injury of iliac vascular and its branches, injury of bladder and urethra, and injury of anus and intestine. We investigated whether there was a difference in the incidence of combined injuries among the four groups. The volume of blood transfusion, the length of hospital stay, the number of operations, the cost of hospitalization, the main complications after discharge, and follow‐up time were determined for the 39 patients who survived. The Majeed pelvic fracture score was used for follow‐up evaluation, as shown in Tables 1 and 2 13.

**Table 1:** Majeed pelvic fracture rating rules

Pain (30 points)	
Intense, continuous at rest	0–5
Intense with activity	10
Tolerable, but limits activity	15
With moderate activity, abolished by rest	20
Mild, intermittent, normal activity	25
Slight, occasional, or no pain	30
Work (20 points)	
No regular work	0–4
Light work	8
Change of job	12
Same job, reduced performance	16
Same job, same performance	20
Sitting (10 points)	
Painful	0–4
Painful if prolonged or awkward	6
Uncomfortable	8
Free	10
Sexual intercourse (4 points)	
Painful	0–1
Painful if prolonged or awkward	2
Uncomfortable	3
Free	4
Standing (36 points)	
A Walking aids (12 points)	
Bedridden or almost	0–2
Wheelchair	4
Two crutches	6
Two sticks	8
One stick	10
No sticks	12
B Gait unaided (12 points)	
Cannot walk or almost	0–2
Shuffling small steps	4
Gross limp	6
Moderate limp	8
Slight limp	10
Normal	12
C Walking distance (12 points)	
Bedridden or few meters	0–2
Very limited time and distance	4
Limited with sticks, difficult without	
Prolonged standing possible	6
One hour with a stick and limited without	8
One hour without sticks slight pain or limp	10
Normal for age and general condition	12

**Table 2:** Clinical grading standard

Working before injury	Not working before injury	Grade
>85	>70	Excellent
70 to 84	55 to 69	Good
55 to 69	45 to 54	Fair
<55	<45	Poor

Clinical grade based on a score out of 100 points for working and 80 points for non‐working patients

The diagnostic criteria for shock were as follows: (i) there were causes of shock; (ii) conscious disturbance; (iii) pulse speed was more than 100 beats per minute or unpalpable; (iv) the limbs were wet and cold, the skin finger pressure on the sternum was positive (the filling time was more than 2 s after compression), the mucosa was pale or cyanotic, and the urine volume was less than 30 mL/h or there was no urine; (v) the systolic blood pressure was lower than 10.7 kPa (80 mm Hg); (vi) the pulse pressure difference was less than 2.7 kPa (20 mm Hg); and (vii) in patients with original hypertension, systolic blood pressure decreased by more than 30% compared with the original level. Where item (i) above is complied with, two of items (ii), (iii), and (iv) and one of items (v), (vi), and (vii) indicated a diagnosis of shock. The injuries of iliac vessels observed in this paper include the injury of internal and external iliac arteries and their branches, and injury of internal and external iliac veins and their branches. At the time of emergency treatment, there was obvious active bleeding in the pelvis, which was addressed first, and the iliac vessel injury was diagnosed after intraoperative exploration and interventional therapy. Diagnostic criteria for bladder and urethra injury: (i) digital rectal examination – in addition to a general physical examination, for patients with posterior urethral injury caused by pelvic fracture, digital rectal examination can be used to determine whether there is a posterior urethral injury and whether there is a rectal injury; (ii) catheterization examination – under strict aseptic conditions, a soft silicone catheter can be inserted to clarify the injury; and (iii) urethrography – urethrography is performed if necessary to determine the location and the extent of the urethral injury. According to the medical history, bleeding, contamination, as well as the position and posture of the injury, combined with local signs and finger examination, diagnosis of anal canal and rectal injuries can be made. Visual examination was done to see if there was fecal contamination. Anal finger examination revealed whether there was anal relaxation or poor sphincter contractile function. If necessary, rectal injuries can be diagnosed by barium enema and endoscopy.

## Statistical Analysis

All statistical analyses are performed using the SPSS statistics software version 22.0 (SPSS, Chicago, IL). To determine whether there is a difference in mortality among subtypes and whether there are differences in complications among subtypes, the Fisher exact test was used. The correlation between each subtype and mortality was analyzed using the correlation analysis of two ordered categorical variables and gamma coefficient γ, in which α = 0.05 test level. Independent *Z*‐test was used to determine whether the subtypes were independent or not. α = 0.05 test level; *P* < 0.05 was considered to be statistically significant.

## Results

### *General Results and Follow‐up Status*

There were 28 deaths, with a mortality rate of 41.8%; 16 patients died within 3 h of admission, 13 with hemorrhagic shock and 3 with craniocerebral injury. Among these, 5 died less than 1 h after admission, 6 died within 1 to 2 h after admission, and 5 died within 2 to 3 h after admission. A total of 12 patients were hospitalized for more than 3 h and died, including 8 from severe infection and 4 from multi‐organ failure. Results of treatment: 39 patients survived; the average blood transfusion volume was 8220 mL/patient (3200–9600 mL); the average hospitalization duration was 128 days/patient (53–174 days); and the average number of surgeries was 5 times/patient (2–8 times). Compared to the average hospitalization cost of a general surgery patient, the cost was 3–5 times more per patient. The main complications were: 3 cases of permanent bladder fistula, 5 cases of permanent intestinal fistula, and 15 cases of infection. The average follow‐up time was 6 months (3 months to 1 year after discharge). The Majeed pelvic fracture score was excellent in 12 cases, good in 14 cases, fair in 9 cases, and poor in 4 cases; there was an excellent and good rate of 66.7%.

### *Mortality Rate and Combined Injury of Regional Injury Classification*

**Figure 1:**
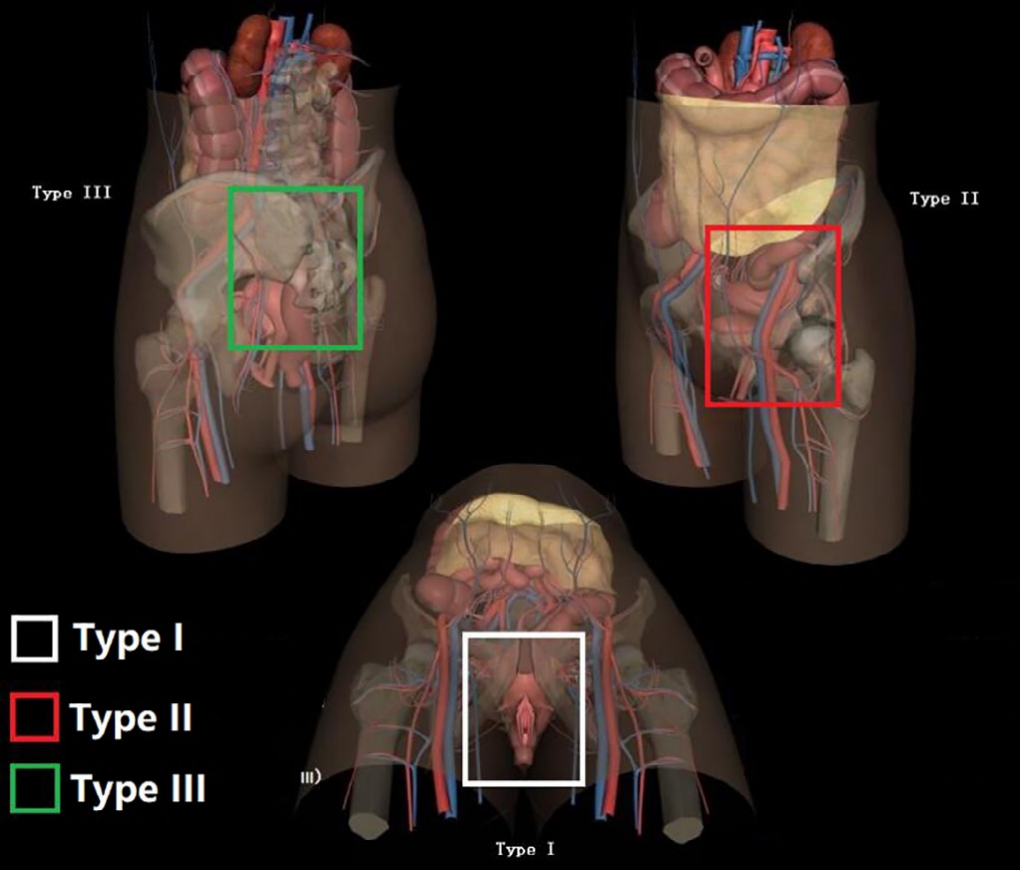
Diagram of regional injury classification in open pelvic fracture. The white frame indicates the perineal type (type I), the red frame indicates ilioinguinal type (type II), and the green frame indicates sacral ilium (type III). The composite type (type IV): Sacroiliac‐perineal type (type III + type I) and ilioinguinal‐perineal type (type II + type I).

Combined with Faringer division, the soft tissue of this group was evaluated using the Faringer classification^9^. The mortality and combined injury of 67 patients with open pelvic fractures was assessed and a new classification according to regional injury classification of open pelvic fracture was proposed (Fig. 1).

**Figure 2:**
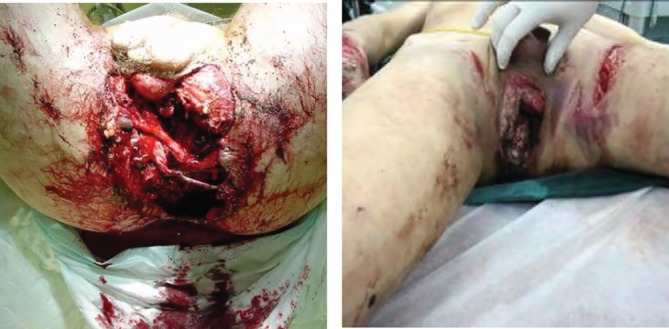
The perineal type (type I). The damage in this area includes the pubis and the ischium region, the perineum, and the pelvic floor region. These cases are type I perineal type, and the perineal area and basin area are seriously injured.

The perineal type (type I): Includes the pubis and the ischium region, the perineum, and the pelvic floor region. There were 29 cases, with 6 deaths accounting for 20.7% (6/29), including urinary tract, genitals, pelvic floor, ischium, pubis, and symphysis pubis injuries. There were 29 cases of shock, 4 cases of iliac vascular injury, 25 cases of bladder urethral injury (86.2% or 25/29), and 15 cases of anorectal injury (51.7% or 15/29) (Fig. 2).

**Figure 3:**
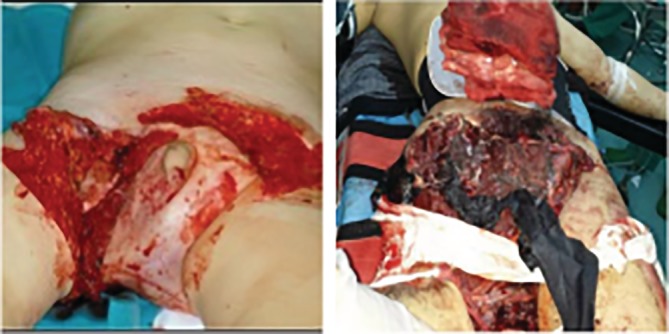
The ilioinguinal type (type II). The damaged area includes the ilioinguinal region, the lower abdomen, and the acetabular anterior 1/2 portion. These cases are type II ilioinguinal type, with severe injuries to the ilioinguinal region and the lower abdomen.

The ilioinguinal type (type II): Includes the ilioinguinal region, the lower abdomen, and the acetabular anterior 1/2 portion. There were 20 cases, including 9 deaths, and iliopubic or/and hip, sacroiliac, iliofemoral vessels, bladder, and intestinal injuries. There were 20 cases of shock, 18 cases of iliac vascular injury, 8 cases of bladder urethral injury, and 5 cases of anorectal injury (Fig. 3).

**Figure 4:**
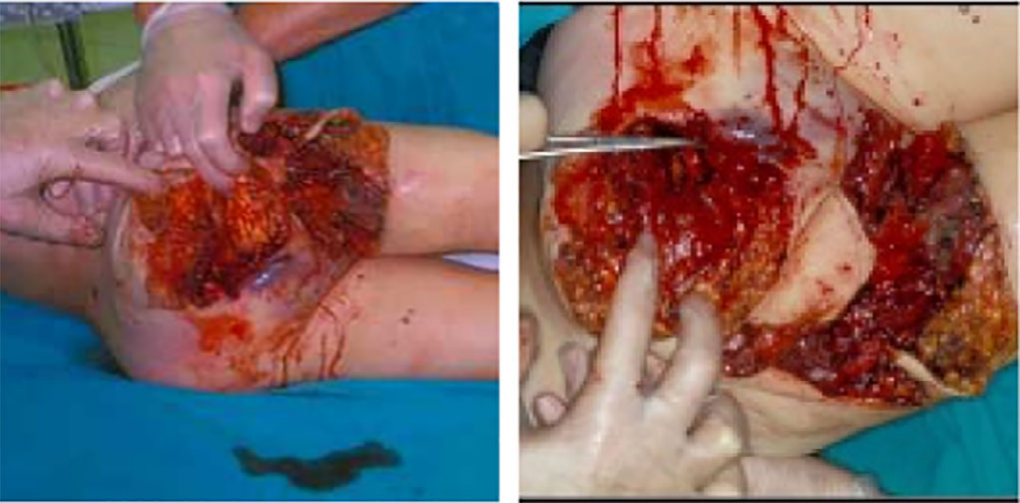
The sacroiliac type (Type III). The damaged area includes the acetabular posterior 1/2 portion, the sacroiliac joint, and the sacrum region. These cases are type III sacroiliac type, with severe injuries to the sacroiliac joint and the sacrum region.

The sacroiliac type (type III): Includes the acetabular posterior 1/2 portion, sacroiliac joint, and sacrum region. Among 7 cases, 5 patients died, accounting for 71.4% (5/7), with sacroiliac joint fracture and dislocation, iliac blood vessel, ureter, and intestinal injuries. There were 7 cases of shock, 7 cases of iliac vascular injury, 1 case of bladder urethral injury, and 2 cases of anorectal injury (Fig. 4).

The composite type (type IV): 11 cases; 8 cases died, accounting for 72.7% (8/11). The Sacroiliac‐perineal type (type III + type I): 6 cases; 5 cases died, accounting for 83.3% (5/6); 6 cases of shock; 6 cases of iliac vascular injury; 6 case of bladder urethral injury; and 2 cases of anorectal injury. The ilioinguinal‐perineal type (type II + type I): 5 cases; 3 cases died, accounting for 60% (3/5); 5 cases of shock; 5 cases of iliac vascular injury; 2 case of bladder urethral injury; and 5 cases of anorectal injury.

### *Statistical Results and Significance*

Through the Fisher exact test of multiple independent samples, with a level of α = 0.05, *P* = 0.006 < 0.05, the perineum type, the ilioinguinal type, and the sacroiliac type were determined to be different or not identical, with statistical significance. There was a positive correlation between classification and mortality, and the degree of correlation was large. The correlation coefficient gamma coefficient, γ, was 0.620, *P* = 0.001 < 0.05, which was statistically significant. The four subtypes were independent of each other, *P* = 0.000 < 0.05, and the difference was statistically significant. The mortality of open pelvic fractures can be evaluated by classification. The mortality rate of type I, type II, type III, and type IV are increased. The severity of open pelvic fractures is also increased by type.

Type I is mainly bladder or urethral injury (86.2%) or anorectal injury (51.7%). Types II (90%) and III (100%) are mainly iliac vascular injuries. Among the type IV composite type, the sacroili–perineal type (type III + type I) is dominated by iliac vascular injury and bladder and urethral injury, while the ilioinguinal‐perineal type (type II + type I) is dominated by iliac vascular injury and direct anal injury. The combined injury incidence of type I and type II open pelvic fractures was 82.8% (24/29) and 85.0% (17/20), respectively. The combined injury incidence of type III and type IV fractures was 100.0% (7/7, 11/11), and the difference was statistically significant (*P* = 0.041). The combined injury incidence of type III and type IV was significantly higher than that of type I and type II (*P* < 0.05) (Table 3).

**Table 3:** Frequency of death and combined injury in open pelvic fractures

Group names	Survival condition		Occurrence of combined injury (case)			
	Survival/Dead	Total	Shock	Iliac vascular injury	Bladder urethral injury	Anorectal injury
The perineal type (I)	23/6	29	29	4	25	15
The ilioinguinal type (II)	11/9	20	20	18	8	5
The sacroiliac type (III)	2/5	7	7	7	1	2
Type IV (type III + I)	1/5	6	6	6	6	2
(Type II + I)	2/3	5	5	5	2	5
The composite type (IV)	3/8	11	11	11	8	7
Total	67		67	40	42	29

## Discussion

### *Treatment Characteristics and Prognosis of Subtypes*

Open pelvic fractures are often multiple injuries, serious injuries, and difficult to treat. The causes of high mortality are hemorrhage as a result of early vascular injury of pelvic organs and infection in middle and late stage of disease^14^. Effective hemostasis is the key to early treatment of open pelvic fractures^15^. For type I, macrovascular injury is less likely, and wound tamponade is the main treatment. Types II and III have high probabilities of macrovascular and vascular plexuses injury; vascular repair and tamponade should be conducted. In type IV, there is a high rate of damage to large vessels, vascular plexuses, and viscera; immediate exploratory laparotomy is required to repair blood vessels and viscera tissues. Positive pelvic gauze packing hemostasis or temporary abdominal aortic occlusion, as well as pelvic angiography and embolization, are effective hemostasis methods. Application of minimally invasive preperitoneal packing balloon and abdominal aortic junction tourniquet has been proposed as an alternative to open preperitoneal packing (OP) for the treatment of bleeding associated with open pelvic fractures^16^. At the same time, most patients also need early transcatheter arterial embolization (TAE) to control definite bleeding^17^. In extreme cases, a half hip or half pelvic amputation is performed. The contamination of the open pelvic fracture wound itself, together with the contamination of the rectum, the anal canal and the bladder, can aggravate the degree of contamination of soft tissues. Retroperitoneal hematoma is a good culture medium for bacteria^5^. Therefore, infection and its complications are the main causes of death in the middle and late stage of open pelvic fractures. Both types I and IV have high risk of mid‐to‐late infection and related complications. Jones reported that the mortality rate of patients with open pelvic fractures accompanied by rectal injury was 45%, and 70% of the patients had systemic infection^7^. Raffa and Christensen reported that early colon ostomy distal rectal debridement reduced the mortality rate of open pelvic fractures from 58% to 25%, and this view was widely accepted^18^. For types I and IV, besides paying attention to wound surface treatment, early sigmoidotomy and cystostomy are the key to treatment. Also important are repeated debridement, thorough drainage, and adequate and combined use of antibiotics. Wound sealing and negative pressure drainage can make wound peripheral tissue move toward the center, and coordinate with the force of fibroblasts to reduce the wound surface. Negative pressure can significantly increase microcirculation flow and microvascular diameter, promote blood circulation, increase cell division, promote the growth of fibroblasts, and, to some extent, promote the healing of the wound surface and the growth of granulation tissue^19^. For type II, attention should be paid to bladder and intestinal injury; early and timely cystostomy is the focus of management. In open pelvic fractures the opening of the wound reduces the pelvic tamponade. The bleeding of the wound is not easily self‐coagulated as a result of peritoneal fibrosis, which greatly increases the risk of bleeding. Temporary fixation of a pelvic binding belt or C‐shaped forceps provides a means of pelvic volume control, which can not only quickly and effectively restore the stability of the pelvic ring but also stabilize pelvic pressure to achieve the effect of hemostasis^20^, ^21^.

### *Advantages and Disadvantages of Regional Injury Classification*

**Figure 5:**
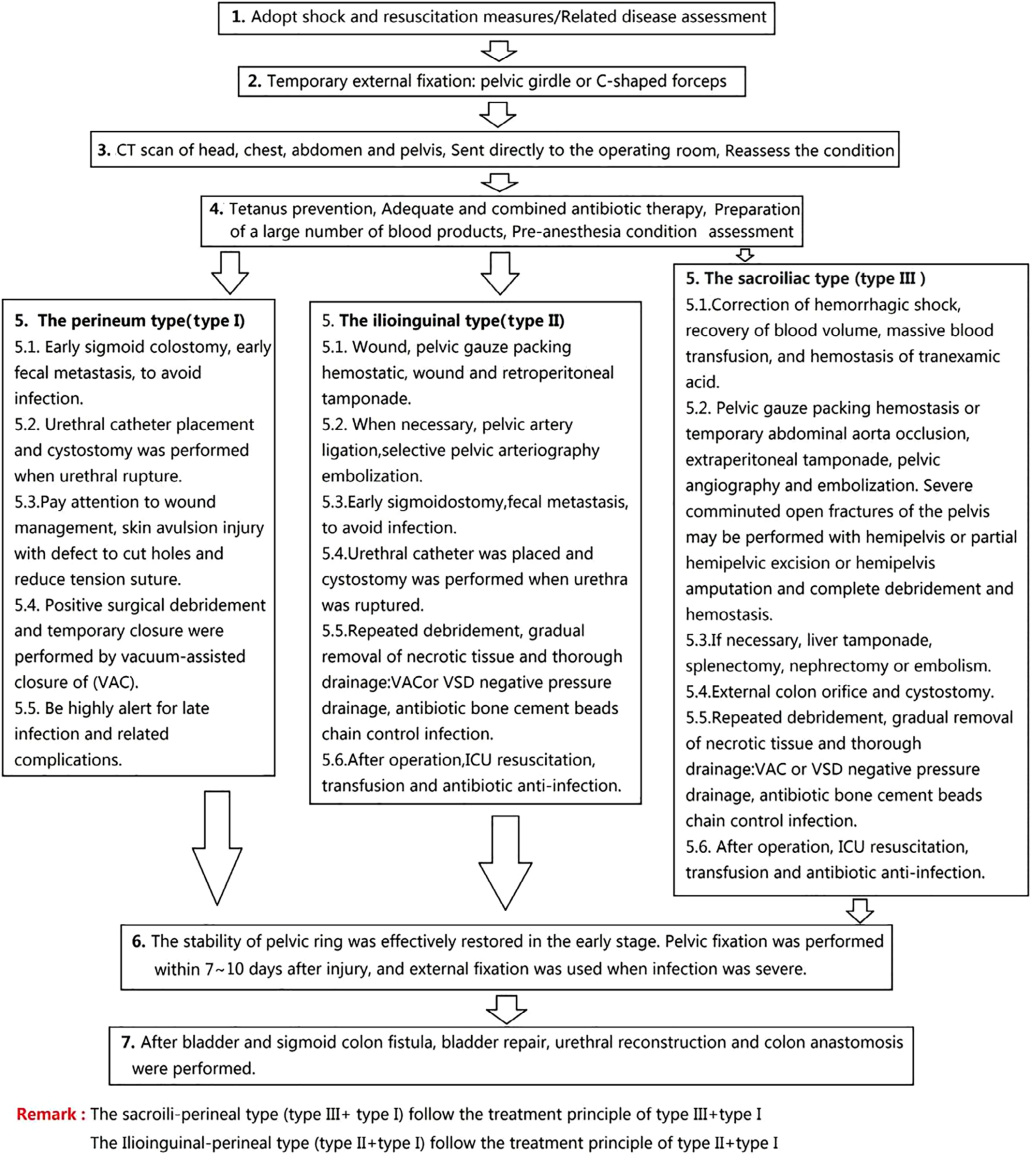
Flow chart of open pelvic fracture treatment.

According to the regional injury classification of open pelvic fractures combined with ATLS guidelines^22^, we developed an open pelvic fracture treatment flow chart (Fig. 5). This treatment flow chart indicates the effective injury control measures for different types of open pelvic fractures. The first time to deal with the most lethal factors of the combined injury and complications, so that the treatment of the process of simplification, standardization, in order to achieve the best treatment effect. A disadvantage of this classification is that there are many factors affecting the mortality of open pelvic fractures, which are not only related to injury division but also related to fracture type, complications, and associated injuries. This paper only discusses the relationship between injury division and mortality. To make the classification more accurate, we also comprehensively considered the occurrence of complications under each classification, including iliac vessel, urethra, and anal injuries and infection and other indicators to further verify the accuracy of the classification.

### *The Shortcomings of this Study*

This classification method only indicates the location and the area of the injury, and not the degree of soft tissue injury. A careful physical examination must be conducted as part of the emergency treatment, and the whole body condition evaluated with Gustilo–Anderson classification and ISS trauma score. After emergency treatment, X‐ray, CT and Tile classification were used to evaluate the condition of the pelvis. The classification of injuries in the open pelvic fracture area did not include the classification of closed pelvic fractures, and could not reflect the stability of pelvic fractures. The author believes that the classification of open pelvic fractures should be conducted in emergency treatment. The most important emergency treatment for open pelvic fractures is early treatment of the causes and complications leading to the highest mortality. The placement of internal fixation can be guided by Tiles classification in the middle and late stages of open pelvic fractures. In the classification of open pelvic fracture area injuries, the type IV composite injuries also include ilioinguinal–perineal–sacroiliac injuries, which often belong to completely separate injuries. The emergency treatment is amputation, which is not included in the open pelvic fracture area injury classification. In addition, specific types of open injuries, such as bullet wounds and stab wounds, were not included in this study. Further research could verify the accuracy of the classification by taking into account factors such as bone fracture classification, shock condition, physical examination, and complications. At the same time, the regional injury and classification system could be combined with Tile, Young–Burgess, occupational therapists association, and other bone fracture classification systems to diagnose open pelvic bone fractures.
