# ERRATUM

**DOI:** 10.1111/os.12661

**Published:** 2020-04-28

In [1], the order of authors in the author byline was incorrectly published as Zheng‐hao Wang, Kai‐nan Li, MM.

The author byline order should be “Kai‐nan Li, MM and Zheng‐hao Wang.”

The publisher apologizes for the error and any convenience it may have caused.
